# The effectiveness of ergonomic intervention for preventing work-related musculoskeletal disorders in agricultural workers: A systematic review protocol

**DOI:** 10.1371/journal.pone.0288131

**Published:** 2023-07-03

**Authors:** Ann Jirapongsuwan, Piyanee Klainin-Yobas, Wanpen Songkham, Siriporn Somboon, Napamon Pumsopa, Patraporn Bhatarasakoon

**Affiliations:** 1 Department of Public Health Nursing, Faculty of Public Health, Mahidol University, Bangkok, Thailand; 2 Alice Lee Center for Nursing Studies, Yong Loo Lin School of Medicine, National University of Singapore, Singapore, Singapore; 3 The Thailand Centre for Evidence Based Health Care: A JBI Affiliated Group, Faculty of Nursing, Chiang Mai University, Chiang Mai, Thailand; 4 Department of Community Health Nursing, Faculty of Nursing, Srinakharinwirot University, Nakhon Nayok, Thailand; Drexel University, UNITED STATES

## Abstract

**Objective:**

This review will systematically examine and synthesize existing evidence of the effectiveness of ergonomic interventions in preventing work-related musculoskeletal disorders in agricultural workers.

**Introduction:**

Agricultural workers are at particular risk of developing musculoskeletal disorders due to the nature of their activities and work conditions. Ergonomic interventions designed to prevent work-related musculoskeletal disorders among agricultural workers can benefit their health and productivity.

**Inclusion criteria:**

The review will consider quantitative study designs. These studies should be about agricultural workers who were involved in occupational situations that might contribute to musculoskeletal disorders.

**Methods:**

The databases including PubMed, CINAHL, Cochrane Central Register of Controlled Trials, Scopus and grey literature will be searched to identify published and unpublished studies reported in English and other languages from 1991 onwards. At least two independent reviewers will screen titles and abstracts and assess the selected full texts against certain inclusion criteria. The identified studies will be assessed for methodological quality using JBI critical appraisal instruments. Data will be extracted, and the effectiveness of the interventions will be determined. Where possible, data will be pooled in a meta-analysis. Data from heterogeneous studies will be reported narratively. The GRADE approach will be used to assess the quality of evidence.

**Systematic review registration number:** PROSPERO CRD42022321098

## Introduction

Work-related musculoskeletal disorders (WMSDs) are injuries or disorders of the musculoskeletal system and connective tissues resulting from an imbalance between work conditions and physical capacities [1]. Work conditions that may lead to WMSDs include the routine lifting of heavy objects, daily exposure to whole body vibration, routine overhead work, work with the neck in chronic flexion position, and repetitive forceful tasks [2]. The duration of exposure (including the number of activities or total exposure time) is also an important factor in the development of such problems. Most WMSDs are cumulative disorders, and symptoms may vary from discomfort and pain to decreased body function and invalidity, which can lead to reduced productivity, sickness, and chronic occupational disability.

Nearly one-third of the world’s workforce is employed in the agricultural sector [3]. The agricultural workforce includes waged agricultural workers, self-employed farmers, and other self-employed workers. Agriculture is a dangerous sector, with a high incidence of occupational diseases and injuries related to the safety and health conditions of workers [3]. Agricultural work varies across different contexts, such as mechanized and manual operations [4, 5]. The International Labour Office and the International Ergonomics Association indicated that the ergonomic-related health conditions of agricultural workers are related to not only their types of operation but also multiple aspects of workplace conditions, such as material storage and handling, work design, and physical conditions [6]. Therefore, the present study will consider including all contexts of agricultural work to review ergonomic interventions concerning such aspects.

Agricultural workers have a high risk of developing WMSDs due to the nature of their activities and extremely labor-intensive workload [7]. In 2016, the World Health Organization reported that the global burden of disease attributable to occupational ergonomic factors was 12.27 million disability-adjusted life-years, an increase of 20.1% from 2000 [8]. The three main WMSD risk factors of agricultural workers, which are involved in every process of their work activities, are the lifting and carrying of different weights, repetitive whole-body work, and handwork [9–11]. Their use of incorrect techniques, awkward positions, prolonged static postures, use of inadequate equipment and tools, and long work durations also increase their risk of exposure [9–11]. The negative impacts of WMSDs on agricultural workers are substantial and result in various symptoms of musculoskeletal problems, including pain and discomfort, long-term disability, and reduced quality of life [2, 12, 13]. The direct and indirect costs related to these problems have been considered in many countries [2]. Given the negative impact of WMSDs, the prevention of WMSDs among agricultural workers has become a worldwide priority.

An imbalance between the load and functional capacity of workers results in the risk of WMSDs. Ergonomics is the interaction between workers and the elements in their working environments; the work and capacity of workers should be balanced by adapting their work to them or developing their capacity [2]. Ergonomic interventions include physical, organizational, and cognitive components [14]. Ergonomic interventions in the physical domain include improving equipment and work environments. Ergonomic interventions in the organizational domain consider work structures and processes. Ergonomic interventions in the cognitive domain consider human cognitive and mental abilities. Ergonomics is adopted to establish a healthy relationship between humans and their working environments, thereby reducing risk, preventing health problems, and increasing worker productivity. Ergonomic interventions are a major way to prevent and control WMSDs [2].

Systematic and scoping reviews about ergonomics in agriculture have been performed. These reviews provide evidence of risk factors regarding musculoskeletal disorders (MSDs) and other health effects [15–17]. A review was conducted to identify ergonomic approaches to WMSDs prevention [18]. However, this study did not define such interventions and the outcomes of the included studies. The results of the studies included ergonomic approaches targeting back stress, body flexion, and work productivity. Therefore, ergonomic interventions for agricultural workers have not been addressed, which underscores the need for a systemic review of ergonomic interventions for the prevention of WMSDs in agriculture.

Preliminary searches of ergonomic interventions for WMSDs have been conducted on different databases, such as the JBI Database of Systematic Reviews and Implementation Reports, PROSPERO, PubMed, and Cochrane Database on Systematic Reviews. Existing evidence of ergonomic interventions concerning WMSDs prevention mostly focuses on workplace settings [14, 19–21]. Studies on ergonomic interventions for agricultural workers emphasize cognitive ergonomic interventions focusing on the knowledge, perception, or behavior of workers [22, 23]. Some of these studies recommend physical activity or exercise interventions to decrease the muscle pain of workers [24]. Physical ergonomic interventions regarding equipment design have also been examined to reduce the musculoskeletal problems of agricultural workers [25, 26]. In addition, a work environment intervention was conducted to reduce the musculoskeletal pain of workers [27].

Ergonomic interventions should prevent MSDs related to the work processes, conditions, and environments in the agricultural setting. No systematic review has been conducted on ergonomic interventions for WMSDs prevention, particularly the effectiveness of various interventions developed to reduce agricultural WMSDs. A well-conducted review should present rigorous evidence of the existence of ergonomic interventions for WMSDs prevention among agricultural workers. Therefore, this systematic review will evaluate the effectiveness of ergonomic interventions for agricultural workers to provide an evidence based basis for preventing WMSDs. This study protocol describes the methods that will be used to locate, select, and critically appraise studies to ensure the quality of the review.

### Review question

The review question is: what is the effectiveness of ergonomic interventions in preventing WMSDs in agricultural workers?

## Methods

The proposed systematic review will be conducted per the Joanna Briggs Institute (JBI) methodology for systematic reviews of effectiveness evidence [28]. The review title has been registered with PROSPERO, and the registration number is CRD42022321098.

### Inclusion criteria

#### Participants

This review will consider studies involving agricultural workers who were in work environments or performed work activities that may contribute to MSDs, such as lifting, pulling, pushing, standing, walking, bending, and repetitive movements. The sociocultural background of the workers will be disregarded.

#### Interventions

This review will evaluate any type of ergonomic intervention designed to prevent WMSDs in agricultural workers. No limitation will be imposed on the geographical region and frequency, intensity, and duration of the interventions and co-interventions. Ergonomic interventions include physical, organizational, and cognitive components [14]. Ergonomic interventions in the physical domain include improvements in equipment and work environments, such as enhancements in equipment designs and workplace layouts. Ergonomic interventions in the organizational domain consider work structures and processes, such as work times/durations, design, and flow. Ergonomic interventions in the cognitive domain consider human cognitive abilities and mental processes, such as knowledge, perception, and skills training.

#### Comparators

This review will consider studies that compared ergonomic interventions with no-intervention controls, alternative interventions, or usual care approaches.

#### Outcomes

This review will include studies that used MSDs as an outcome. The components of MSDs will include pain (e.g., back pain and shoulder pain), symptoms, discomfort, disability (e.g., MRI specific lesion, MRI lesion-specific lesion, Carpal tunnel, or Rotator cuff syndrome), and physical functions (e.g., flexibility and muscle strength). Self-report measures (e.g., dichotomy scale and visual analog scale) and direct measures (e.g., goniometer, flexometer, and dynamometer) will be accepted. Studies will be included if they reported one of the outcomes. No restriction will be imposed on the type of self-report or objective measure used.

#### Types of studies

This review will include randomized and nonrandomized controlled trials, before-and-after studies, and other types of quasi-experimental studies. In addition, analytical observational studies, including prospective and retrospective cohort studies and case-control studies, will be included considered for inclusion. This review will be included studies in English and other languages.

#### Search strategy

The search strategy aims to find published and unpublished studies. A three-phase search strategy will be conducted [28]. In the first phase, an initial keyword search of PubMed, CINAHL, and PROSPERO will be undertaken. Then, the text contained in the titles, abstracts, and index terms will be analyzed to identify relevant articles. In the second phase, each database will be searched for all identified keywords and index terms. In the third phase, the reference lists of all identified studies will be searched for additional relevant studies. The search time frame will be set to 1991 onward to ensure that the studied interventions include vulnerable technologies and practices.

A librarian will be consulted to ensure the relevance of the search terms. A comprehensive search will be conducted, and six electronic databases will be searched for published studies: PubMed, CINAHL, Cochrane Central Register of Controlled Trials, and Scopus. Sources, including ProQuest Dissertations and Theses and Google Scholar will be searched for unpublished studies and grey literature. Cross-checking information sources in grey literature will be conducted. ClinicalTrials.gov and the World Health Organization International Clinical Trials Registry Platform will also be searched. The reference lists of all studies selected for critical appraisal will be manually screened for additional studies. The full search strategies will be provided in the complete review. Table 1 is a sample search strategy for PubMed, including the keywords, which will be used in line with the population, intervention, and outcomes (PICO) framework. Filters will be used to retrieve only full texts published/posted from 1991 onward.

**Table 1:** PubMed search strategy.

PICO	Step	Search Term
Population	#1	agricultural worker* [tw]
	#2	agricultural worker [mh]
	#3	farmer* [tiab]
	#4	farmworker* [tw]
	#5	farmworker [mh]
	#6	farm* [tiab]
	#7	dairy worker* [tiab]
	#8	#1 OR #2 OR #3 OR #4 OR #5 OR #6 OR #7
Intervention	#9	educational intervention* [tw]
	#10	educational intervention [mh]
	#11	physical activity* [tw]
	#12	physical activity [mh]
	#13	ergonomic intervention* [tw]
	#14	ergonomic intervention [mh]
	#15	ergonomic training* [tw]
	#16	ergonomic training [mh]
	#17	exercise intervention* [tw]
	#18	exercise intervention [mh]
	#19	exercise program* [tiab]
	#20	work environment* [tw]
	#21	work environment [mh]
	#22	#9 OR #10 OR #11 OR #12 OR #13 OR #14 OR #15 OR #16 OR #17 OR #18
Outcome	#23	musculoskeletal problem* [tw]
	#24	musculoskeletal disorders* [tw]
	#25	musculoskeletal disorders [mh]
	#26	musculoskeletal symptom* [tw]
	#27	musculoskeletal pain* [tw]
	#28	muscle pain* [tw]
	#29	myalgia [mh]
	#30	back pain* [tw]
	#31	backache* [tw]
	#32	shoulder pain* [tw]
	#33	musculoskeletal discomfort* [tw]
	#34	physical function* [tiab]
	#35	muscle flexibility* [tiab]
	#36	muscle strength* [tiab]
	#37	#20 OR #21 OR #22 OR #23 OR #24 OR #25 OR #26 OR #27 OR #28 OR #29 OR #30 OR #31 OR #32 OR #33 OR #34 OR #35 OR #36
#38		#8 AND #22 AND #37
Filter		1991–2022

#### Study selection

The study selection process will follow the Preferred Reporting Items for Systematic Reviews and Meta-Analysis (PRISMA) flow [29]. Initially, the titles and abstracts of all identified records will be screened against the criteria. The full texts of eligible articles will be downloaded and reviewed independently. The references of these selected articles will be imported into an Excel file to form the list of eligible studies. Duplicate studies will be removed, and two independent reviewers will screen the titles and abstracts based on the review inclusion criteria. Then, two independent reviewers will assess the full texts of the selected citations according to the criteria. The reasons for the exclusion of full text studies will be reported in the systematic review. Any disagreement will be resolved through a discussion with a third reviewer. The search results will be reported in the final systematic review.

#### Assessment of methodological quality

Eligible studies will be critically appraised by two independent reviewers for methodological validity before their inclusion in the review using standardized critical appraisal instruments from the JBI [30]. Two reviewers will perform a pilot test on two studies to facilitate agreement between the reviewers. Any disagreement between the reviewers will be resolved through a discussion, possibly including a third reviewer. The authors of research articles will be contacted if the published articles have missing information or report only summary data. Following the critical appraisal, sensitivity analyses will be conducted and high-quality studies will be included in this review.

#### Data extraction

Data will be extracted from the included studies by two independent reviewers using the standardized data extraction tool in JBI System for the Unified Management of the Assessment and Review of Information (JBI-SUMARI) [30]. The data extracted will include specific details about the authors, years, interventions, populations, study methods, and outcomes of significance to the review question and specific objectives. Any disagreement between the reviewers will be resolved through a discussion, which may include a third reviewer. The data extraction form will undergo pilot testing to ensure agreement between the two reviewers. Study authors will be contacted electronically to obtain missing data or additional data if needed.

#### Data synthesis

Where possible, quantitative data will be pooled in a meta-analysis using JBI-SUMARI. All results will be subject to double data entry. Effect sizes, expressed as risk ratios (for categorical data) or standardized mean differences (for continuous data), and their 95% confidence intervals (CIs) will be calculated for analysis. Heterogeneity will be assessed statistically using the standard chi-squared and I-squared tests (I-squared < 50% indicates low heterogeneity, and I-squared ≥ 50% indicates high heterogeneity). The results of comparable groups of studies will be pooled using a fixed-effects model, and their 95% CIs will be calculated. A random-effects model will be used if high heterogeneity exists between studies. Mean differences with 95% CIs will be used for continuous variables. Standardized mean differences and 95% CIs will be used for continuous variables with different units. Then, the meta-analysis will be conducted. If statistical pooling is not possible, the findings will be presented in a narrative form, and figures will be used for data presentation where appropriate. Publication bias will be examined using funnel plots if 10 or more studies are included in the meta-analysis. Depending on data availability, subgroup analysis will be performed to compare the effects of interventions per intervention type [14], geographical region, gender, main study design or type of agricultural work (e.g., mechanized operation and manual operation). Sensitivity analysis may be performed to identify potential outliers and/or assess the robustness of effect sizes to review outcomes. The results will be combined to form a conclusion.

#### Assessment of quality of findings

The Grading of Recommendations Assessment, Development, and Evaluation (GRADE) approach will be used to grade the quality of evidence in this review. The software GRADEPro will be used to summarize the findings. The summary will present information about appropriate absolute risks for treatment and control, estimates of relative risk, and a ranking of evidence quality based on the risk of bias, indirectness, inconsistency, imprecision, and publication bias of the review results. Two independent reviewers will assess each study, and any disagreement will be resolved through a discussion. The evidence quality results will be presented under four categories: high, moderate, low, and very low [31].

## Discussion

The agricultural sector has a high rate of WMSDs. Agricultural workers are at risk of such health problems because of many of their tasks and activities, which depend on their work locations, environmental conditions, and types of machinery and tools. Systematic reviews of ergonomic studies for agricultural workers have been performed to prevent WMSDs, but these works focus mostly on MSD risk factors and health effects. This study will be a significant contribution to the literature by providing the best available evidence of the effectiveness of ergonomic interventions in preventing WMSDs in agricultural workers, which has not been studied. The systematic review design aims to obtain robust, conclusive evidence to help enhance the quality of studies on this issue. This systematic review will be published in a peer-reviewed journal. Any significant change to this protocol will be noted with a description and the rationale of the change.

## Supporting information

S1 Checklist PRISMA-P 2015 checklist. (DOCX)

S1 File A sample search strategy for PubMed. (DOCX)
