# Supplementary material for: The effectiveness of ergonomic intervention for preventing work-related musculoskeletal disorders in agricultural workers: A systematic review protocol
Source: PLoS One. 2023 Jul 3;18(7):e0288131. doi: 10.1371/journal.pone.0288131 (PMC10317214; doi:10.1371/journal.pone.0288131)
Supplement: S1 File — (DOCX) [file pone.0288131.s002.docx]

**S2 A sample search strategy for PubMed**

Search commenced on PubMed (Search date: May 19, 2023)

| **Database** | **Step** | **Search Terms** | **Number of studies** |
| --- | --- | --- | --- |
| Population | #1 | agricultural worker* [tw] | 7,499 |
|  | #2 | agricultural worker [mh] | 3,943 |
|  | #3 | farmer* [tiab] | 29,248 |
|  | #4 | farmworker* [tw] | 1,238 |
|  | #5 | farmworker [mh] | 3,943 |
|  | #6 | farm* [tiab] | 114,376 |
|  | #7 | dairy worker* [tiab] | 111 |
|  | #8 | #1 OR #2 OR #3 OR #4 OR #5 OR #6 OR #7 | 119,425 |
| Intervention | #9 | educational intervention* [tw] | 14,800 |
|  | #10 | educational intervention [mh] | 38,790 |
|  | #11 | physical activity* [tw] | 146,498 |
|  | #12 | physical activity [mh] | 244,525 |
|  | #13 | ergonomic intervention* [tw] | 550 |
|  | #14 | ergonomic intervention [mh] | 2,088 |
|  | #15 | ergonomic training* [tw] | 118 |
|  | #16 | ergonomic training [mh] | 7,150 |
|  | #17 | exercise intervention* [tw] | 8,942 |
|  | #18 | exercise intervention [mh] | 20,685 |
|  | #19 | exercise program*[tiab] | 16,923 |
|  | #20 | work environment*[tw] | 14,086 |
|  | #21 | work environment [mh] | 153 |
|  | #22 | #9 OR #10 OR #11 OR #12 OR #13 OR #14 OR #15 OR #16 OR #17 OR #18 OR #19 OR #20 OR #21 | 423,554 |
| Outcome | #23 | musculoskeletal problem* [tw] | 1,657 |
|  | #24 | musculoskeletal disorders* [tw] | 9,422 |
|  | #25 | musculoskeletal disorders [mh] | 1,203,580 |
|  | #26 | musculoskeletal symptom* [tw] | 2,405 |
|  | #27 | musculoskeletal pain* [tw] | 10,547 |
|  | #28 | muscle pain* [tw] | 4,777 |
|  | #29 | myalgia [mh] | 2,651 |
|  | #30 | back pain* [tw] | 73,731 |
|  | #31 | backache* [tw] | 3,985 |
|  | #32 | shoulder pain* [tw] | 11,270 |
|  | #33 | musculoskeletal discomfort* [tw] | 434 |
|  | #34 | physical function* [tiab] | 33,027 |
|  | #35 | muscle flexibility* [tiab] | 187 |
|  | #36 | muscle strength* [tiab] | 30,713 |
|  | #37 | #23 OR #24 OR #25 OR #26 OR #27 OR #28 OR #29 OR #30 OR #31 OR #32 OR #33 OR #34 OR #35 OR #36 OR #37 | 1,318,014 |
| #38 | | #8 AND #22 AND #35 | 95 |
| Filter | | 1991-2022 | 92 |

Note:

tw = Text Word

tiab = Title/Abstract

mh = MeSH Terms

Initial keyword will be used to be:

(("agricultural worker*"[Text Word] OR "farmer*"[Title/Abstract] OR "farmworker*"[Text Word] OR "farmers"[MeSH Terms] OR "farm*"[Title/Abstract] OR "dairy worker*"[Title/Abstract] OR "farmers"[MeSH Terms]) AND ("musculoskeletal problem*"[Text Word] OR "musculoskeletal disorders*"[Text Word] OR "musculoskeletal diseases"[MeSH Terms] OR "musculoskeletal symptom*"[Text Word] OR "musculoskeletal pain*"[Text Word] OR "muscle pain*"[Text Word] OR "myalgia"[MeSH Terms] OR "back pain*"[Text Word] OR "backache*"[Text Word] OR "musculoskeletal discomfort*"[Text Word] OR "physical function*"[Title/Abstract] OR "muscle flexibility*"[Title/Abstract] OR "muscle strength*"[Title/Abstract] OR "shoulder pain*"[Text Word]) AND ("educational intervention*"[Text Word] OR (("educability"[All Fields] OR "educable"[All Fields] OR "educates"[All Fields] OR "education"[MeSH Subheading] OR "education"[All Fields] OR "educational status"[MeSH Terms] OR ("educational"[All Fields] AND "status"[All Fields]) OR "educational status"[All Fields] OR "education"[MeSH Terms] OR "education s"[All Fields] OR "educational"[All Fields] OR "educative"[All Fields] OR "educator"[All Fields] OR "educator s"[All Fields] OR "educators"[All Fields] OR "teaching"[MeSH Terms] OR "teaching"[All Fields] OR "educate"[All Fields] OR "educated"[All Fields] OR "educating"[All Fields] OR "educations"[All Fields]) AND "methods"[MeSH Terms]) OR "physical activity*"[Text Word] OR "exercise"[MeSH Terms] OR "ergonomic intervention*"[Text Word] OR (("ergonomical"[All Fields] OR "ergonomically"[All Fields] OR "ergonomics"[MeSH Terms] OR "ergonomics"[All Fields] OR "ergonomic"[All Fields]) AND "methods"[MeSH Terms]) OR "ergonomic training*"[Text Word] OR (("ergonomical"[All Fields] OR "ergonomically"[All Fields] OR "ergonomics"[MeSH Terms] OR "ergonomics"[All Fields] OR "ergonomic"[All Fields]) AND "education"[MeSH Terms]) OR "exercise intervention*"[Text Word] OR (("exercise"[MeSH Terms] OR "exercise"[All Fields] OR "exercises"[All Fields] OR "exercise therapy"[MeSH Terms] OR ("exercise"[All Fields] AND "therapy"[All Fields]) OR "exercise therapy"[All Fields] OR "exercise s"[All Fields] OR "exercised"[All Fields] OR "exerciser"[All Fields] OR "exercisers"[All Fields] OR "exercising"[All Fields]) AND "methods"[MeSH Terms]) OR "exercise program*"[Title/Abstract] OR "work environment*"[Text Word] OR "working conditions"[MeSH Terms])) AND (1991:2022[pdat])

Appendix II: Data extraction instruments

JBI Critical Appraisal Checklist for quasi-experimental studies

Reviewer ______________________________________ Date_______________________________

Author_______________________________________ Year_________ Record Number_________

|  | Yes | No | Unclear | Not applicable |
| --- | --- | --- | --- | --- |
| 1. Is it clear in the study what is the ‘cause’ and what is the ‘effect’ (i.e. there is no confusion about which variable comes first)? | □ | □ | □ | □ |
| 1. Were the participants included in any comparisons similar? | □ | □ | □ | □ |
| 1. Were the participants included in any comparisons receiving similar treatment/care, other than the exposure or intervention of interest? | □ | □ | □ | □ |
| 1. Was there a control group? | □ | □ | □ | □ |
| 1. Were there multiple measurements of the outcome both pre and post the intervention/exposure? | □ | □ | □ | □ |
| 1. Was follow up complete and if not, were differences between groups in terms of their follow up adequately described and analyzed? | □ | □ | □ | □ |
| 1. Were the outcomes of participants included in any comparisons measured in the same way? | □ | □ | □ | □ |
| 1. Were outcomes measured in a reliable way? | □ | □ | □ | □ |
| 1. Was appropriate statistical analysis used? | □ | □ | □ | □ |

Overall appraisal: Include □ Exclude □ Seek further info □

Comments (Including reason for exclusion)

JBI Critical Appraisal Checklist for randomized Controlled trials

Reviewer ______________________________________ Date_______________________________

Author_______________________________________ Year_________ Record Number_________

|  | Yes | No | Unclear | NA |
| --- | --- | --- | --- | --- |
| 1. Was true randomization used for assignment of participants to treatment groups? | □ | □ | □ | □ |
| 1. Was allocation to treatment groups concealed? | □ | □ | □ | □ |
| 1. Were treatment groups similar at the baseline? | □ | □ | □ | □ |
| 1. Were participants blind to treatment assignment? | □ | □ | □ | □ |
| 1. Were those delivering treatment blind to treatment assignment? | □ | □ | □ | □ |
| 1. Were outcomes assessors blind to treatment assignment? | □ | □ | □ | □ |
| 1. Were treatment groups treated identically other than the intervention of interest? | □ | □ | □ | □ |
| 1. Was follow up complete and if not, were differences between groups in terms of their follow up adequately described and analyzed? | □ | □ | □ | □ |
| 1. Were participants analyzed in the groups to which they were randomized? | □ | □ | □ | □ |
| 1. Were outcomes measured in the same way for treatment groups? | □ | □ | □ | □ |
| 1. Were outcomes measured in a reliable way? | □ | □ | □ | □ |
| 1. Was appropriate statistical analysis used? | □ | □ | □ | □ |
| 1. Was the trial design appropriate, and any deviations from the standard RCT design (individual randomization, parallel groups) accounted for in the conduct and analysis of the trial? | □ | □ | □ | □ |

Overall appraisal: Include □ Exclude □ Seek further info □

Comments (Including reason for exclusion)

_______________________________________________________________________________________________________________________________________________________________________________________________________________________________________________________________________________________

JBI Critical Appraisal Checklist for case control studies

Reviewer ______________________________________ Date_______________________________

Author_______________________________________ Year_________ Record Number_________

|  | Yes | No | Unclear | Not applicable |
| --- | --- | --- | --- | --- |
| 1. Were the groups comparable other than the presence of disease in cases or the absence of disease in controls? | □ | □ | □ | □ |
| 1. Were cases and controls matched appropriately? | □ | □ | □ | □ |
| 1. Were the same criteria used for identification of cases and controls? | □ | □ | □ | □ |
| 1. Was exposure measured in a standard, valid and reliable way? | □ | □ | □ | □ |
| 1. Was exposure measured in the same way for cases and controls? | □ | □ | □ | □ |
| 1. Were confounding factors identified? | □ | □ | □ | □ |
| 1. Were strategies to deal with confounding factors stated? | □ | □ | □ | □ |
| 1. Were outcomes assessed in a standard, valid and reliable way for cases and controls? | □ | □ | □ | □ |
| 1. Was the exposure period of interest long enough to be meaningful? | □ | □ | □ | □ |
| 1. Was appropriate statistical analysis used? | □ | □ | □ | □ |

Overall appraisal: Include □ Exclude □ Seek further info □

Comments (Including reason for exclusion)

_______________________________________________________________________________________________________________________________________________________________________________________________________________________________________________________________________________________

JBI Critical Appraisal Checklist for cohort studies

Reviewer ______________________________________ Date_______________________________

Author_______________________________________ Year_________ Record Number_________

|  | Yes | No | Unclear | Not applicable |
| --- | --- | --- | --- | --- |
| 1. Were the two groups similar and recruited from the same population? | □ | □ | □ | □ |
| 1. Were the exposures measured similarly to assign people to both exposed and unexposed groups? | □ | □ | □ | □ |
| 1. Was the exposure measured in a valid and reliable way? | □ | □ | □ | □ |
| 1. Were confounding factors identified? | □ | □ | □ | □ |
| 1. Were strategies to deal with confounding factors stated? | □ | □ | □ | □ |
| 1. Were the groups/participants free of the outcome at the start of the study (or at the moment of exposure)? | □ | □ | □ | □ |
| 1. Were the outcomes measured in a valid and reliable way? | □ | □ | □ | □ |
| 1. Was the follow up time reported and sufficient to be long enough for outcomes to occur? | □ | □ | □ | □ |
| 1. Was follow up complete, and if not, were the reasons to loss to follow up described and explored? | □ | □ | □ | □ |
| 1. Were strategies to address incomplete follow up utilized? | □ | □ | □ | □ |
| 1. Was appropriate statistical analysis used? | □ | □ | □ | □ |

Overall appraisal: Include □ Exclude □ Seek further info □

Comments (Including reason for exclusion)

_____________________________________________________________________________________________
